# Complete Genome Sequence of Lactococcus lactis subsp. *lactis* G121, an Isolate with Allergy-Protective Features Derived from a Farming Environment

**DOI:** 10.1128/MRA.00777-20

**Published:** 2020-10-08

**Authors:** Zhongjie Wang, Eva-Maria L. Minarsch, Susanne Kublik, Holger Heine, Michael Schloter, Baerbel U. Foesel

**Affiliations:** a Research Unit for Comparative Microbiome Analysis, Helmholtz Center Munich, German Research Center for Environmental Health, Neuherberg, Germany; b Division of Innate Immunity, Research Center Borstel, Airway Research Center North, German Center for Lung Research, Borstel, Germany; c Technical University of Munich, Central Institute for Food and Health, Freising, Germany; Queens College

## Abstract

Early childhood exposure to a farming environment has been found to be protective against asthma and other atopic disorders. Here, we report the complete genome sequence of Lactococcus lactis subsp. *lactis* G121, which was isolated from the kitchen of a farm in Bavaria (Germany) and is recognized for its allergy-protective properties. It could be assembled into one circular chromosome, three circular plasmids, and one linear plasmid.

## ANNOUNCEMENT

Lactococcus lactis has been described as an abundant member of the cowshed microflora (1) which is considered an important component for the protective effect of farming environments in early childhood, significantly decreasing the risk of developing atopic disorders later in life (2, 3). A subsequent analysis of an isolate derived from a farm, namely, Lactococcus lactis subsp. *lactis* G121, revealed a reduction of allergic reactions in sensitized mice, and the strain was able to activate mammalian cells *in vitro* and to induce a TH1-polarizing program in dendritic cells (1).

Here, we present the complete genome of L. lactis subsp. *lactis* G121, which was isolated from a farmer’s kitchen in Bavaria, Germany (1). Genome sequencing was carried out on the PacBio Sequel platform (Pacific Biosciences, Menlo Park, CA) using v2 chemistry. Library preparation was performed according to the procedure and checklist for preparing multiplexed microbial SMRTbell libraries for the PacBio Sequel system. Briefly, DNA from cells grown overnight in liquid LB at 30°C was extracted using the Genomic-tip 20/G kit (Qiagen, Hilden, Germany). To increase the extraction efficiency, a preincubation of the bacterial culture with ampicillin (0.6 μg⋅liter^−1^) for 3 h prior to cell harvesting was included. Genomic DNA was sheared to approximately 10 kb using g-TUBEs (Covaris, Inc., Woburn, MA) and, without additional size selection, further processed according to the protocol. The library including L. lactis subsp. *lactis* G121 was loaded onto two single-molecule real-time (SMRT) cells at concentrations of 3 pM and 6 pM, according to the diffusion loading protocol (PacBio). The movie time was 10 h per SMRT cell after immobilization for 2 h and preextension for 2 h. After data demultiplexing, genome assembly for L. lactis subsp. *lactis* G121 (using combined data from the two SMRT cells) was performed with the Microbial Assembly pipeline embedded in SMRT Link v8.0.0.80529 (PacBio) (parameters deviating from default settings were as follows: seed coverage, 45; set genome size, 2.7 Mb), utilizing 194,273 realigned subreads, with an average subread length of 3,534 bp. The mean coverage was 253-fold, and the genome contained five scaffolds after initial assembly, including one linear chromosome, three circular plasmids, and one linear plasmid. The linear chromosomal scaffold was circularized successfully using Circlator v1.5.5 with the parameter --merge_min_length_merge 1000 (4). Coding sequence (CDS), rRNA, and tRNA genes were scanned and annotated with the NCBI Prokaryotic Genome Annotation Pipeline (PGAP) (5). The genome was also functionally annotated using the RAST server v2.0 (6).

The overall genome had a size of 2,712,646 bp (GC content, 35%) and contained 2,783 predicted CDSs. As shown in Table 1, the genome consisted of a chromosome of 2,573,315 bp (GC content, 35.18%) and four plasmids. Plasmid.001F, containing 69,239 bp (GC content, 31.36%), was still linear. The other three plasmids, containing 42,612 bp (GC content, 31.8%), 11,744 bp (GC content, 36.54%), and 9,908 bp (GC content, 33.62%), could be circularized.

**TABLE 1:** Genome features of Lactococcus lactis subsp. *lactis* G121

Name	Contig length (bp)	GC content (%)	Topology
Chromosome.000F	2,573,315	35.18	Circular
Plasmid.001F	69,239	31.36	Linear
Plasmid.002F	42,612	31.80	Circular
Plasmid.003F	11,744	36.55	Circular
Plasmid.004F	9,908	33.62	Circular

Nineteen rRNA genes were annotated by PGAP, including six 16S rRNA genes. In addition, 65 tRNAs were detected, but only 1 transfer-messenger RNA gene was found. Based on the RAST annotation, 27% of the CDSs could be assigned to specific subsystems, including the categories carbohydrates (210 CDSs), amino acids and derivatives (188 CDSs), protein metabolism (123 CDSs), and cofactors, vitamins, prosthetic groups, and pigments (120 CDSs). Within the carbohydrate subsystem category, 40 genes were assigned to fermentation, which is closely related to the described phenotypic properties of this species.

### Data availability.

The complete genome sequence of Lactococcus lactis subsp. *lactis* G121 has been deposited in GenBank under accession numbers CP053671 to CP053674 and CP061027. The raw reads have been deposited in the Sequence Read Archive under BioProject accession number PRJNA633429.
